# A Comparison of Bioimpedance Spectroscopy or Tape Measure Triggered Compression Intervention in Chronic Breast Cancer Lymphedema Prevention

**DOI:** 10.1089/lrb.2021.0084

**Published:** 2022-12-15

**Authors:** Sheila H. Ridner, Mary S. Dietrich, John Boyages, Louise Koelmeyer, Elisabeth Elder, T. Michael Hughes, James French, Nicholas Ngui, Jeremy Hsu, Vandana G. Abramson, Andrew Moore, Chirag Shah

**Affiliations:** ^1^ Vanderbilt University School of Nursing, Nashville, Tennessee, USA.; ^2^ Department of Biostatistics, Vanderbilt University School of Medicine, Nashville, Tennessee, USA.; ^3^ Australian Lymphoedema Education, Research, and Treatment Program, Macquarie University, Macquarie Park, Australia.; ^4^ Department of Clinical Medicine, Macquarie University, Macquarie Park, Australia.; ^5^ Medicine, Health & Human Sciences, ICON Cancer Center, Wahroonga, Australia.; ^6^ Westmead Breast Cancer Institute, Westmead Hospital, Westmead, Australia.; ^7^ The University of Sydney, Sydney, Australia.; ^8^ Lakeside Specialist Breast Clinic, Lakeview Private Hospital, Norwest, Australia.; ^9^ Northern Surgical Oncology, Sydney Adventist Hospital, Wahroonga, Australia.; ^10^ Sydney Adventist Hospital Clinical School, College of Health and Medicine, Australian National University, Acton, Australia.; ^11^ Division of Hematology/Oncology, Vanderbilt-Ingram Cancer Center, Nashville, Tennessee, USA.; ^12^ Southeast Cancer Center, Cape Girardeau, Missouri, USA.; ^13^ Cleveland Clinic, Taussig Cancer Institute, Cleveland, Ohio, USA.

**Keywords:** breast cancer, lymphedema, prevention, tape measure, bioimpedance spectroscopy

## Abstract

**Background:**

This study compared rates of progression to chronic breast cancer-related lymphedema (defined as ≥ 10% arm volume change from baseline requiring complex decongestive physiotherapy [CDP]) following an intervention for subclinical lymphedema (S-BCRL) triggered by bioimpedance spectroscopy (BIS) or by tape measurement (TM).

**Methods and Results:**

This stratified, randomized, international trial enrolled new breast cancer patients undergoing: mastectomy/partial mastectomy, axillary treatment (dissection, sentinel lymph node biopsy [SLNB] >6 nodes or radiation), radiation therapy (chest wall/breast, supraclavicular fossa), or taxane-based chemotherapy. Following postsurgery eligibility reassessment, centralized, 1:1 randomization to prospective surveillance by BIS or TM occurred. S-BCRL detection triggered a 4-week, 12-hour per day, compression sleeve, and gauntlet intervention. The primary outcome (*n* = 209), rates of postintervention progression to CDP, was assessed over 3 years. Between June 24, 2014 and September 11, 2018, 1200 patients were enrolled, 963 randomized (BIS *n* = 482; TM *n* = 481) and 879 analyzed (BIS *n* = 442; TM *n* = 437). Median follow-up was 32.9 months (interquartile range = 22, 35). BIS patients triggered an intervention at a lower rate than TM patients (20.1%, *n* = 89 vs. 27.5%, *n* = 120, *p* = 0.011). Median months to trigger were longer with BIS than TM (9.7; 95% confidence interval [CI], 8.2–12.6 vs. 3.9; 95% CI, 2.8–4.5, *p* = 0.001). Overall, 14.4% (*n* = 30) progressed post-intervention, with reduced likelihood for BIS patients than TM patients (7.9%, *n* = 7 vs. 19.2%, *n* = 23; relative risk = 0.41; 95% CI, 0.13–0.81; absolute reduction 11.3%; 95% CI, 2.3–20.3; *p* = 0.016).

**Conclusions:**

Compared to TM, BIS provides a more precise identification of patients likely to benefit from an early compression intervention.

Clinical Trial Registration number: NCT02167659.

## Introduction

As breast cancer survivorship rises, the impact of long-term treatment complications has taken on greater significance.^1^ One such complication is breast cancer-related lymphedema (BCRL).^2–4^ Chronic BCRL (C-BCRL) can lead to pain, infections, limited arm function, reduced quality of life, and costly and resource intensive therapies, such as complex decongestive physiotherapy (CDP).^5,6^ Rates of C-BCRL range from 5% with breast conserving surgery with SLNB alone to greater than 50% with axillary lymph node dissection (ALND), regional node irradiation (RNI), and/or taxane-based chemotherapy.^5^

Identifying patients in the early stage of BCRL can be challenging. Currently, a diagnosis of C-BCRL is usually made after visible/clinically apparent changes or symptoms occur.^7–9^ Before these changes, subclinical disease occurs as indicated by an increase in extracellular fluid.^10^ Accurately identifying subclinical disease before it is visible facilitates early intervention and possibly C-BCRL prevention.^10^

Bioimpedance spectroscopy (BIS) allows for early identification of subclinical extracellular fluid change.^11,12^ Early diagnosis of subclinical BCRL (S-BCRL) coupled with short-course compression therapy has been shown to improve outcomes, suggesting that such early detection may prevent the need for CDP.^13–17^ The primary aim of this study was to determine if subclinical detection of increasing extracellular fluid through BIS and early intervention with 4 weeks of a compression sleeve and gauntlet resulted in reduced rates of progression to C-BCRL, defined as ≥10% arm volume change from baseline requiring CDP, compared to the same intervention when initiated by increasing arm volume ascertained using circumferential tape measurement (TM).

## Materials and Methods

### Study design

This multicenter, international, randomized clinical trial (RCT) compared BIS and TM measurements for BCRL surveillance among newly diagnosed breast cancer patients. When patients in either group exceeded pre-established change thresholds from baseline a compression intervention was triggered. The trial was conducted in breast clinics at four sites in Australia and nine sites in the United States. Study procedures were initially approved by the Vanderbilt University Institutional Review Board (IRB) and the Vanderbilt Ingram Center Scientific Review Committee (SRC). Subsequent approvals were obtained by individual site IRBs and when required institutional SRCs. Vanderbilt University School of Nursing (VUSN) served as the coordinating/lead site, provided all training, as well as data review, maintenance, and analysis.

The Research Electronic Data Capture (REDCap) database environment was used for data collection and management.^18^ Fidelity oversight visits and data/safety audits were conducted annually at each of the sites.

### Participants

Presurgical inclusion criteria included women over 18 years old with histologically confirmed newly diagnosed breast cancer (invasive carcinoma or ductal carcinoma *in situ* [DCIS]) with a planned surgical procedure. Exclusion criteria included: history of breast cancer; neoadjuvant chemotherapy (latter half of recruitment phase of study only as 3 years follow-up would extend past planned study end); previous radiation to the breast/chest wall/or axilla for any reason; active implanted medical device (e.g., pace maker); medical conditions known to cause swelling; pregnancy; previous arm lymphedema treatment; uncontrolled intercurrent illness; psychiatric illness that would limit compliance; planned bilateral surgery; or an allergy to electrode adhesives or compression fabrics.

The study was designed to be reflective of an elevated risk of S-BCRL. Therefore, eligibility was re-evaluated 2 months (±5 days) after surgery before randomization, with inclusion criteria of stage I–III invasive breast cancer or DCIS with at least one of the following: mastectomy, axillary treatment (ALND, SLNB with >6 nodes removed), or RNI to the chest wall/breast, axilla, and/or supraclavicular fossa, or taxane-based chemotherapy. Those undergoing bilateral mastectomies were excluded. Written informed consent was obtained from all participants.

### Procedures

Baseline presurgical measurements (including both BIS and TM) were completed for all enrolled patients. Patients who remained eligible after the second evaluation were immediately randomized with 1:1 allocation, using a computer-generated, permuted block program (blocks of four) to measurement by either BIS or TM. Centralized randomization, stratified by site, was implemented by VUSN. Due to the difference in appearance of a BIS device or TM, blinding was not possible for the research assistants taking measurements.

Those assigned to the BIS group were assessed using an L-Dex^®^U400 (ImpediMed Limited, Brisbane, Australia). Measurements were conducted by trained research staff following manufacturer's instructions and reported in L-Dex units. Those in the TM group were measured with a Gulick II tape. Using a marked board to facilitate correct tape placement, arms were measured twice at 10 cm increments from the wrist up to 50 cm above the wrist by trained research staff. Arm volume was auto-calculated using a truncated cone formula, and the average of the two assessments was used for evaluation.

Initially the intervention trigger for the BIS group was ≥10 L-Dex units in the absence of a >10% volume change from pretreatment baseline and for the TM group an at-risk arm volume change of ≥5% and <10% compared to baseline and contralateral measurements. In either group a volume change of >10% resulted in direct referral for CDP. In 2016, published studies demonstrated the presence of early-stage C-BCRL with the BIS of seven rather than 10 L-Dex units.^19–21^ We verified those findings in a sample of 280 women.^22^ Thus, with IRB and SRC approval the intervention trigger was modified from ≥10 L-Dex units to ≥6.5 L-Dex units for all previously enrolled and subsequent patients assigned to the BIS group.

Postoperative BIS or TM assessments for all end points were at 3, 6, 12, 18, 24, and 36 months and following compression intervention. Optional visits at 15 and 21 months were allowed at site PI discretion. Patients that met the intervention trigger for S-BCRL wore a class 2 (23–32 mmHg, medi flat knit custom or Harmony^®^ circular knit) compression sleeve and gauntlet for 4 weeks, 12 hours/day.

When a participant triggered an intervention, they underwent the alternative measurement, ensuring that both BIS and TM measurements were taken for all participants before intervention initiation. Regardless of group assignment, if at intervention triggering, intervention completion, or any measurement post-intervention TM volume in the at-risk arm was ≥10% change from baseline (absent a similar change in the non-at-risk arm) the patient was referred for CDP due to BCRL progression and removed from the study.

### Statistical analysis

We hypothesized that compared to TM detection of subclinical swelling, BIS detection would reduce the rate of lymphedema progression requiring CDP by as much as 20%. Estimates of progression vary widely; thus, a rate of 50% using TM was used to determine that group sizes of 100 achieved 80% statistical power to detect at least that much difference (two sided <0.05). If the observed rates in the TM group were higher or lower than 50%, then the statistical power of a proposed 20% difference would increase and smaller differences could be detected.

Categorical variables were summarized using frequency distributions and compared using chi-square tests of independence. Median and interquartile range were used to summarize continuous study variables; group comparisons were conducted using Mann–Whitney tests.

Analyses of progression after triggering used intent-to-treat (ITT) principles (comparison of randomization conditions regardless of whether or not patients completed intervention post-trigger). The primary hypothesis was that surveillance and early detection using the BIS triggering standard as compared to the standard using TM surveillance would result in lower rates of C-BCRL. C-BCRL was defined as ≥ 10% arm volume change from baseline. The test of differences between the study group in progression to C-BCRL post-trigger and intervention was conducted using logistic regression. A bootstrapped probability and 95% confidence interval (CI) were generated around the parameter estimate using 1000 bootstrap samples. Statistical significance was established as a maximum two-sided Type I error of 0.05.

## Results

**FIG. 1.**
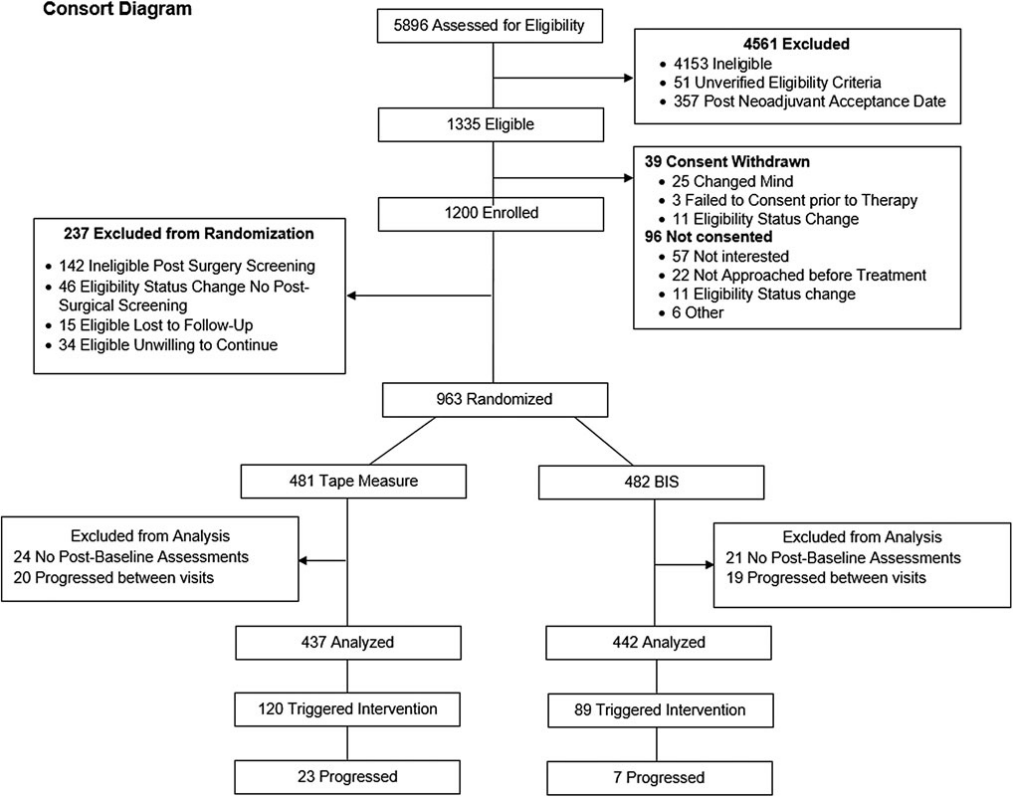
Consort diagram.

Overall, 1239 women were recruited between June 24, 2014 and September 11, 2018 with 1200 enrolled; postoperatively, 963 patients met the inclusion criteria and were randomized (BIS *n* = 482: TM *n* = 481) (see Fig. 1, Consort diagram). The final analysis was performed upon completion of the study (December 31, 2020) on 879 patients (BIS *n* = 442; TM *n* = 437) as 39 (4.2%) patients progressed either at their initial postrandomization visit or between other study visits (BIS *n* = 19; TM *n* = 20, *p* = 0.85) and 45 had no valid postbaseline assessments (BIS *n* = 21; TM *n* = 24, *p* = 0.64). The sample had a median age of 58.5 years with most being non-Hispanic nor Latina (96.4%) (Table 1). With the exception of patients assigned to TM having a slightly higher rate of smoking (37% vs. 30%, *p* = 0.051), the groups were well balanced for demographic and pathologic features.

**Table 1.** Sociodemographic and Environmental Characteristics at Baseline

Characteristic	Overall (*N* = 879)	TM group (*n* = 437)	BIS group (*n* = 442)	*p*
	Median [IQR] (*N*)			
Age	58.5 [50, 67] (878)	58.8 [50, 66] (437)	58.4 [50, 67] (441)	0.944
Missing	1	0	1	
Years of education	16.0 [12, 16] (873)	16.0 [12, 16] (435)	16.0 [12, 16] (438)	0.444
Missing	6	2	4	

	*n* (%)			
Race				0.774
Asian	76 (8.8)	38 (8.7)	38 (8.8)	
Black or African American	67 (7.7)	31 (7.1)	36 (8.3)	
White	678 (78.1)	345 (79.3)	333 (76.9)	
Multiracial or other	47 (5.4)	21 (4.8)	26 (6.0)	
Missing	11	2	9	
Ethnicity				0.685
Non-Hispanic nor Latina	805 (96.4)	399 (96.1)	406 (96.7)	
Hispanic or Latina	30 (3.6)	16 (3.9)	14 (3.3)	
Missing	44	22	22	
Ever smoked	294 (33.5)	160 (36.6)	134 (30.4)	0.051
Ever drank alcohol	621 (70.7)	315 (72.1)	306 (69.4)	0.380
Stage of cancer				0.058
0 (DCIS)	54 (6.1)	27 (6.2)	27 (6.1)	
I	496 (56.4)	228 (52.2)	268 (60.6)	
II	270 (30.7)	152 (34.8)	118 (26.7)	
III	59 (6.7)	30 (6.9)	29 (6.6)	

	Median [IQR]			
L-Dex units	0.1 [−3.1, 3.2]	0.1 [−3.2, 3.2]	0.2 [−3.0, 3.2]	0.999
Arm volume				
At-risk arm (mL)	1966.4 (1694, 2354)	1968.7 [1692, 2342]	1966.4 [1697, 2369]	0.775
Non-at-risk arm (mL)	1970.0 [1689, 2371]	1964.5 [1688, 2351]	1973.9 [1689, 2392]	0.613
Percent difference	0.1 [−3.1, 3.2]	0.2 [−3.2, 3.2]	0.1 [−2.8, 3.2]	0.672
BMI	27.9 [24.6, 32.5]	28.3 [24.6, 32.5]	27.6 [24.5, 32.4]	0.684

All participants indicated female gender.

BIS, bioimpedance spectroscopy; BMI, body mass index; DCIS, ductal carcinoma *in situ*; IQR, interquartile range; TM, tape measurement.

Clinical characteristics also were well balanced, with the group of BIS patients having a slightly higher prevalence of Stage I disease than TM (61% vs. 52%). In terms of treatment, the BIS group was composed of a slightly higher proportion of patients who had SLNB than the TM group (84% vs. 78%, *p* = 0.011; Table 2). No other statistical or meaningful differences in number of nodes removed, utilization of RNI, or taxane-based chemotherapy were observed between groups. Furthermore, within the ultimate sample (those who triggered an intervention) for testing our primary outcome (progression), no significant or meaningful differences between the rates of ALND (30.3% vs. 27.6%, *p* = 0.127) or cancer stage at presentation were observed (*p* = 0.475; Table 3).

**Table 2.** Breast Treatment Characteristics of all Randomized Participants

	Overall (*N* = 879)	TM group (*n* = 437)	BIS group (*n* = 442)	*p*
Treatment characteristics				
	*n* (%)			
Type of surgery				0.209
Breast conservation	683 (77.9)	329 (75.5)	354 (80.3)	
Mastectomy	178 (20.3)	99 (22.7)	79 (17.9)	
Both	16 (1.8)	8 (1.8)	8 (1.8)	
Missing	2	1	1	
Axillary surgery				0.011
ALND	149 (17.4)	83 (19.4)	66 (15.5)	
SLNB only	692 (81.0)	334 (78.0)^a^	358 (84.0)^b^	
Other	13 (1.5)	11 (2.6)^a^	2 (0.5)^b^	
Missing	25	9	16	
If, SLNB only				0.884
≤6 nodes	675 (97.7)	325 (97.6)	350 (97.8)	
>6 nodes	16 (2.3)	8 (2.4)	8 (2.2)	
Missing	1	1	0	

	Median [IQR] (Min, Max, *N*)			
Total number of nodes dissected	3.0 [2, 4] (0, 63, 855)	3.0 [2, 4] (0, 63, 428)	3.0 [2, 4] (1, 45, 427)	0.153
ALND	16.0 [6, 25] (2, 63, 149)	16.0 [6, 25] (2, 63, 83)	16.5 [5, 25] (2, 45, 66)	0.635
SLNB only	2.0 [2, 4] (0, 15, 691)	3 [2, 3] (0, 11, 333)	2.0 [2, 4] (1, 15, 358)	0.642
Total number of positive nodes	0.0 [0, 0] (0, 31, 855)	0.0 [0, 0] (0, 31, 428)	0.0 [0, 0] (0, 24, 427)	0.138
ALND	2.0 [0, 4] (0, 31, 149)	2.0 [0, 4] (0, 31, 83)	2.0 [0, 4] (0, 24, 66)	0.916
SLNB only	0.0 [0, 0] (0, 3, 691)	0.0 [0, 0] (0, 3, 333)	0.0 [0, 0] (0, 2, 358)	0.450

		*n* (%), *N*		
Any positive nodes	200 (23.4), 855	108 (25.2), 428	92 (21.5), 427	0.203
ALND	105 (70.5), 149	58 (69.9), 83	47 (71.2), 66	0.859
SLNB only	92 (13.3), 691	47 (14.1), 333	45 (12.6), 358	0.550

	*n* (%)			
Chemotherapy	(*N* = 878)	(*N* = 436)	(*N* = 442)	0.997
None	514 (58.5)	255 (58.5)	259 (58.6)	
Neoadjuvant only	35 (4.0)	18 (4.1)	17 (3.8)	
Adjuvant	299 (34.1)	148 (33.9)	151 (34.2)	
Both	30 (3.4)	15 (3.4)	15 (3.4)	
Missing	1	1	0	
If chemo, type				0.439
Any taxane	321 (88.2)	162 (89.5)	159 (86.9)	
Other (nontaxane)	43 (11.8)	19 (10.5)	24 (13.1)	
Radiation therapy				
None	144 (16.4)	80 (18.3)	64 (14.5)	0.125
Radiation fields				
Breast/chest wall	725 (99.9)	352 (99.7)	373 (100.0)	0.304
Regional nodes	152 (20.9)	78 (22.1)	74 (19.8)	0.455
SCF	115 (75.7)	58 (74.4)	57 (77.0)	0.702
ICF	13 (8.6)	8 (10.3)	5 (6.8)	0.441
IMC	69 (45.4)	34 (43.6)	35 (47.3)	0.646
Axilla level 1	56 (36.8)	34 (43.6)	22 (29.7)	0.077
Axilla level 2	29 (19.1)	17 (21.8)	12 (16.2)	0.382
Axilla level 3	22 (14.5)	13 (16.7)	9 (12.2)	0.430
Missing	9	4	5	
Endocrine therapy	667 (76.3)	325 (74.7)	342 (77.9)	0.267
Missing	5	2	3	
Complete treatment				0.584
Surgery only	87 (10.0)	50 (11.4)	37 (8.5)	
Surgery+radiation (not RNI)	397 (45.4)	194 (44.4)	203 (46.5)	
Surgery+RNI	33 (3.8)	15 (3.4)	18 (4.1)	
Surgery+chemo (taxane)	54 (6.2)	31 (7.1)	23 (5.3)	
Surgery+chemo (not taxane)	6 (0.7)	2 (0.5)	4 (0.9)	
Surgery+radiation (not RNI)+chemo (taxane)	153 (17.5)	70 (16.0)	83 (19.0)	
Surgery+radiation (not RNI)+chemo (not taxane)	27 (3.1)	13 (3.0)	14 (3.2)	
Surgery+RNI+chemo (taxane)	107 (12.2)	58 (13.3)	49 (11.2)	
Surgery+RNI+chemo (not taxane)	10 (1.1)	4 (0.9)	6 (1.4)	
Missing	5	0	5	
High risk				0.354
ALND or SLNB >6 nodes	27 (3.2)	17 (4.0)	10 (2.4)	
ALND or SLNB >6+RNI	7 (0.8)	3 (0.7)	4 (0.9)	
ALND/SLNB >6+RNI+chemotherapy (taxane)	74 (8.8)	40 (9.5)	34 (8.0)	
ALND/SLNB >6+RNI+chemotherapy (not taxane)	5 (4.4)	1 (0.2)	4 (0.9)	
None of these combinations	731 (86.6)	360 (85.5)	371 (87.7)	
Missing	35	16	19	

All participants had surgery as per inclusion criteria.

_a_ _,_ _b_ Indicate statistically significant pairwise comparisons, Bonferroni-corrected, *p* < 0.05.

ALND, axillary lymph node dissection; SLNB, sentinel lymph node biopsy; RNI, regional node irradiation.

**Table 3.** Critical Clinical and Breast Treatment Characteristics of Sample Triggering Intervention (*N* = 209)

	Overall (*N* = 209)	TM group (*n* = 120)	BIS group (*n* = 89)	*p*
	Median [IQR]			
BMI (baseline)	28.5 [24.0, 34.4]	28.4 [25.0, 34.2]	28.8 [24.9, 35.0]	0.801
Stage of cancer				0.475
0 (DCIS)	109 (52.2)	57 (47.5)	52 (58.4)	
I	68 (32.5)	43 (35.8)	25 (28.1)	
II	26 (12.4)	16 (13.3)	10 (11.2)	
III	6 (2.9)	4 (3.3)	2 (2.2)	

Detailed treatment characteristics				
	*n* (%)			
Type of surgery				0.600
Breast conservation	149 (71.6)	84 (70.6)	65 (73.0)	
Mastectomy	56 (26.9)	34 (28.6)	22 (24.7)	
Both	3 (1.4)	1 (0.8)	(2.2)	
Missing	1	1	0	
Axillary surgery				0.127
ALND	60 (29.1)	36 (30.3)	24 (27.6)	
SLNB only	141 (68.4)	78 (65.5)	63 (72.4)	
Other	5 (2.4)	5 (4.2)	0 (0.0)	
Missing	3	1	2	
If, SLNB only				0.201
≤6 nodes	139 (98.6)	76 (97.4)	63 (100.0)	
>6 nodes	2 (1.4)	2 (2.6)	0 (0.0)	

	Median [IQR] (*N*)			
Total number of nodes dissected	3.0 [2, 9] (206)	3.0 [2, 10] (119)	3.0 [2, 6] (87)	0.416
ALND	19.0 [11, 25] (60)	19.0 [10, 26] (36)	19.5 [12, 24] (24)	0.838
SLNB only	3.0 [2, 4] (141)	3.0 [2,43] (78)	2.0 [2, 4] (63)	0.508
Total number of positive nodes	0.0 [0, 1] (206)	0.0 [0, 2] (119)	0.0 [0, 1] (87)	0.292
ALND	3.0 [1, 8] (60)	3.0 [1, 6] (36)	3.5 [1, 8] (24)	0.909
SLNB only	0.0 [0, 0] (141)	0.0 [0, 0] (78)	0.0 [0, 0] (63)	0.467

		*n* (%), *N*		
Any positive nodes	75 (36.4), 206	47 (39.5), 119	28 (32.2), 87	0.28
ALND	51 (85.0), 60	32 (88.9), 36	19 (79.2), 24	0.31
SLNB only	23 (16.3), 141	14 (17.9), 78	9 (14.3), 63	0.56

	*n* (%)			
Chemotherapy				1.000
None	106 (50.7)	61 (50.8)	45 (50.6)	
Neoadjuvant only	14 (6.7)	8 (6.7)	6 (6.7)	
Adjuvant	77 (36.8)	44 (36.7)	33 (37.1)	
Both	12 (5.1)	7 (5.8)	5 (5.6)	
If chemo, type				0.433
Any taxane	96 (93.2)	54 (91.5)	42 (95.5)	
Other (nontaxane)	7 (6.8)	5 (8.5)	2 (4.5)	
Radiation therapy				
None	34 (16.2)	21 (17.5)	13 (14.6)	0.575
Radiation fields				
Breast/chest wall	172 (99.4)	97 (99.0)	75 (100.0)	0.380
Regional nodes	57 (32.9)	32 (32.7)	25 (33.3)	0.925
SCF	46 (80.7)	27 (84.4)	19 (76.0)	0.427
ICF	7 (12.3)	5 (15.6)	2 (8.0)	0.384
IMC	26 (45.6)	12 (37.5)	14 (56.0)	0.164
Axilla level 1	20 (35.1)	14 (43.8)	6 (24.0)	0.121
Axilla level 2	8 (14.0)	5 (15.6)	3 (12.0)	0.696
Axilla level 3	10 (17.5)	7 (21.9)	3 (12.0)	0.331
Missing	2	1	1	
Endocrine therapy	154 (74.0)	120 (72.5)	67 (76.1)	0.555
Missing	1	0	1	
Complete treatment				0.597
Surgery only	21 (10.1)	14 (11.7)	7 (8.0)	
Surgery+radiation (not RNI)	78 (37.5)	45 (37.5)	33 (37.5)	
Surgery+RNI	7 (3.4)	2 (1.7)	5 (5.7)	
Surgery+chemo (taxane)	16 (7.7)	10 (8.3)	6 (6.8)	
Surgery+chemo (not taxane)	0 (0.0)	0 (0.0)	0 (0.0)	
Surgery+radiation (not RNI)+chemo (taxane)	30 (14.4)	15 (12.5)	15 (17.0)	
Surgery+radiation (not RNI)+chemo (not taxane)	7 (3.4)	5 (4.2)	2 (2.3)	
Surgery+RNI+chemo (taxane)	49 (23.6)	29 (24.2)	20 (22.7)	
Surgery+RNI+chemo (not taxane)	0 (0.0)	0 (0.0)	0 (0.0)	
Missing	1	0	1	
High risk				0.323
ALND or SLNB >6 nodes	4 (2.0)	4 (3.4)	0 (0.0)	
ALND or SLNB >6+RNI	2 (1.0)	1 (0.9)	1 (1.1)	
ALND/SLNB >6+RNI+chemotherapy (taxane)	41 (20.1)	25 (21.4)	16 (18.4)	
ALND/SLNB >6+RNI+chemotherapy (not taxane)	0 (0.0)	0 (0.0)	0 (0.0)	
None of these combinations	157 (77.0)	87 (74.4)	70 (80.5)	
Missing	5	3	2	

All participants had surgery as per inclusion criteria.

Median follow-up was 32.9 months (interquartile range [IQR] = 22, 35); with a trend for longer follow-up in the BIS arm than TM (33.1 months; IQR = 25, 35 vs. 32.7 months; IQR = 20, 35, *p* = 0.054). A lower proportion of BIS patients triggered an intervention (BIS 20.1%, *n* = 89 of 442; 95% CI, 16.3–24.0 vs. TM: 27.5%, *n* = 120 of 437; 95% CI, 22.9–31.7; *p* = 0.011) and median months from randomization to intervention triggering were longer in the BIS group than the TM group (9.7 months; 95% CI, 8.2–12.6 vs. 3.9 months; 95% CI, 2.8–4.5, *p* = 0.001; Table 4). Of the 209 patients, BIS (*n* = 89) and TM (*n* = 120) who triggered an intervention, 30 (14.4%) progressed after intervention. Of those patients, no difference between the groups was observed in intervention completion rates (BIS 92.1%, *n* = 7 vs. TM: 95.0%, *n* = 6; *p* = 0.396).

**Table 4.** Summary of Trigger and Progression by Surveillance Group

	All patients	TM group	BIS group	*p*
	*N* = 918^a^	*N* = 457	*N* = 461	
	*n* (%)	*n* (%)	*n* (%)	
Progressed before intervention	39 (4.2)	20 (4.4)	19 (4.1)	0.848
Sample for potential trigger	*N* = 879	*N* = 437	*N* = 442	

	Median (IQR)	Median (IQR)	Median (IQR)	
Follow-up (months)^b^	32.9 (22.7, 34.3)	32.7 (20.8, 34.3)	33.1 (25.8,34.7)	0.054

	*n* (%)	*n* (%)	*n* (%)	
Triggered	209 (23.8)	120 (27.5)	89 (20.1)	0.011

	Median (IQR)	Median (IQR)	Median (IQR)	
Time from randomization to trigger (months)	5.1 (1.3, 15.6)	3.9 (1.0, 11.6)	9.7 (3.6, 18.2)	0.001
	*N* = 209	*N* = 120	*N* = 89	

	*n* (%)	*n* (%)	*n* (%)	
Progression^c^	30 (14.4)	23 (19.2)	7 (7.9)	0.016^d^

	Median (Min, Max)	Median (Min, Max)	Median (Min, Max)	
Time from trigger to progression (months)	9.6 (0.7, 31.9)	10.7 (1.4, 31.9)	4.9 (0.7, 15.2)	0.100

^a^ Sample with any data post-treatment.

^b^ Months from randomization to last assessment using either tape or BIS.

^c^ Progression to criteria for being off-study without the interim intervention (subclinical) threshold.

^d^ Reduction 11.3%; 95% CI, 2.3–20.3; OR = 0.36; 95% CI, 0.11–0.77, *p* = 0.016, RR = 0.41; 95% CI, 0.13–0.81.

CI, confidence interval; RR, relative risk.

Following intervention, patients in the BIS group were less likely to progress to CDP than those in the TM group (7.9%, *n* = 7 of 89 vs. 19.2%, *n* = 23 of 120; absolute reduction 11.3%; 95% CI, 2.3–20.3; OR = 0.36, 95% CI = 0.11–0.77, *p* = 0.016). Relative rates of progression were reduced by ∼59% in the BIS arm (relative risk = 0.41; 95% CI, 0.13–0.81). Median follow-up months between trigger and progression for the TM or BIS groups were not statistically significantly different (BIS: 4.9 months; 95% CI, 0.7–13.9 vs. TM: 10.7 months; 95% CI, 4.9–17.0, *p* = 0.100; Table 4). An analysis of progression that included only the patients who completed the intervention resulted in similar findings (BIS 8.5%, *n* = 7 of 82 vs. TM: 20.2%, *n* = 23 of 114, absolute reduction 11.7%; 95% CI, 2.1–21.3, *p* = 0.018).

While the randomized groups who triggered were well-balanced in terms of known risk factors for progression to CDP with no statistically significant differences between them, they certainly were not “case/control matched” or “equivalent.” To account for the possibility that one of those factors rather than the triggering approach accounted for the difference in progression rates, risk-adjusted analyses were conducted. As shown in Table 5, while some of those increased the likelihood of progression to CDP (cancer stage, *p* = 0.002; ALND, *p* < 0.001; any positive nodes removed, *p* = 0.007; chemotherapy, *p* = 0.005; a combination of high-risk treatments, *p* = 0.031), after adjusting for those factors the estimates of the effects of using BIS as the BCRL detection method remained essentially equivalent for all adjustments (bootstrapped ORs of 0.35–0.37, with *p*-values remaining between 0.01 and 0.03 for effect of BIS; Table 5).

**Table 5.** Risk Adjusted Progression

	OR	95% CI	*p*
BIS (BMI, *p* = 0.288)	0.36	0.14–0.88	0.017
BIS (cancer stage, *p* = 0.002)	0.35	0.13–0.90	0.022
BIS (mastectomy, *p* = 0.298)	0.36	0.14–0.88	0.014
BIS (ALND, *p* < 0.001)	0.36	0.14–0.92	0.021
BIS (# nodes removed, *p* = 0.002)	0.35	0.14–0.90	0.029
BIS (# positive nodes, *p* = 0.061)	0.36	0.14–0.89	0.023
BIS (any positive nodes, *p* = 0.009)	0.39	0.15–0.96	0.022
BIS (any chemo, *p* = 0.002)	0.35	0.13–0.86	0.018
BIS (if chemo, taxane, *N* = 103, *p* = 0.276)	0.31	0.10–0.92	0.024
BIS (any radiation, *p* = 0.330)	0.36	0.14–0.90	0.019
BIS (if radiation, RNI, *N* = 171, *p* = 0.098)	0.23	0.07–0.71	0.009
BIS (high risk Tx combination, *p* = 0.031)	0.37	0.15–0.93	0.023

Bootstrapped bias-corrected parameter estimates (OR), CIs, and *p*-values for effect of BIS (compared to TM) on the likelihood of progression to CDP after adjusting for the effect of each of the specified known risks for progression. Each of the risks and the probability of their effect on progression that was controlled for in each analysis are shown in ().

CDP, complex decongestive physiotherapy; OR, odds ratio.

Adverse event information was available for the 963 randomized patients. The overall number of study-related adverse events was <1% (*n* = 3). Two reports were of Grade 1 skin itching/tingling/redness during the compression intervention. One patient reported perceived swelling of upper arm during the intervention, but physical examination and measurement did not confirm physiological swelling. There were no study related serious adverse events.

## Discussion

The results from this large, multicenter RCT add to the prior reported data on this topic supporting that use of BIS for prospective BCRL screening coupled with early intervention of a short 4-week well-fitted compression sleeve and gauntlet reduces progression to C-BCRL compared to TM.^23,24^ Specifically, use of BIS as part of prospective BCRL surveillance, coupled with early compression sleeve and gauntlet intervention, significantly reduced C-BCRL (progression to CDP), (7.9% vs. 19.2%, *p* = 0.016) compared to TM.

Analyses including patients who did not complete intervention after triggering, as well as just those who completed intervention, both supported the significant finding. This represents a clinically significant outcome, offering clinicians the ability to use this approach to reduce a patient's risk of developing C-BCRL and potentially affords payers the option of a low-cost, conservative compression sleeve intervention instead of expensive treatment such as physical therapy, lymphatic surgery, and CDP.

This RCT provides level I evidence compared to previous studies with limited numbers of patients that did not use S-BCRL definitions.^12,25^ This is supported by a meta-analysis of more than 67,000 women and 50 studies that found that use of BIS reduced annualized and cumulative incidence of C-BCRL compared to TM or background studies, although not controlling for intervention protocols as the current study did.^26^ These consistent significant findings are likely related to the ability of BIS to detect an increase in extracellular fluid, as opposed to TM's ability to only detect an increase in whole arm volume.

BIS serves as a better screening method to determine who will best benefit from a prevention intervention and achieve reversal of the S-BCRL process that can lead to C-BCRL.^23,24^ BIS is more specific for lymphedema detection than TM as it had fewer triggers and longer times to intervention trigger. The lower rates of C-BCRL in the BIS group would therefore be secondary to more accurate detection and identification of patients with S-BCRL, rather than earlier intervention *per se* as the time to trigger is significantly longer.

A critically important issue regarding the robustness of our findings is that not only did we conduct a rigorous ITT analysis of the randomized groups, we also risk-adjusted for those factors known to increase the likelihood of progression. Randomization insured patients had equal opportunity for both types of assessment; however, “equivalence” of any other factor is not assured. Both groups were well balanced in terms of high-risk factors for progression (no statistically significant differences).

Risk-adjustment analysis confirmed that most of those factors (cancer stage, total number of nodes removed, any positive nodes removed, chemotherapy, and a combination of high-risk treatments) did statistically significantly increase the likelihood of progression. The parameter estimates for the reduction in likelihood of progression with BIS surveillance remained stable (ORs of 0.35–0.37, *p* = 0.01–0.03; Table 5), confirming the balanced nature of the groups. Thus, the statistically and clinically significant positive outcome experienced by patients in the BIS group was related to the screening method and its detection of changes in extracellular fluid.

BIS is clearly a valuable tool for patient management. Our findings are consistent with previous prospective studies which found that use of BIS as part of prospective surveillance resulted in a persistent C-BCRL rate of only 6%.^27^ The final results of the study confirm the significantly lower rates of trigger with BIS compared to TM (20.7% vs. 27.5%, *p* = 0.011) as seen at the interim analysis.^28–30^ Critical knowledge to inform the optimal method of prospective surveillance was also generated in this study. Intensive training, a standardized measurement protocol, and annual fidelity oversight for both measurements were undertaken. While the BIS protocol can be easily replicated in clinical settings, the rigor of the TM protocol for this study exceeded what is practical in most clinics. Thus, BIS may offer even more benefit across clinical settings than what was demonstrated in this study.

The trial was amended during enrollment to reflect a lower L-Dex trigger.^21^ Despite this change, TM still generated more intervention triggers. Although intra- and interobserver variability with TM may have contributed to the greater number of triggers in the TM group, the required training and annual fidelity visits were intended to limit this risk as much as possible. With respect to time to trigger, a difference remained between the two techniques although the difference was smaller than in the interim analysis. This raises questions as to whether TM is capturing postsurgical/radiation inflammatory soft tissue changes that BIS does not capture, rather than lymphedema. If true, then patients may be diagnosed with lymphedema when they do not have it and experience unnecessary psychological distress.

Although this study has many strengths, some limitations do exist. While high-risk features and cancer treatment techniques were well balanced between arms and the statistical analyses controlled for those known and/or potential confounding effects, there may exist effects of those features and/or treatments that could not be accounted for in this trial. Furthermore, this RCT compared BIS to TM and, as such, extrapolation to other BCRL diagnostics is not possible.

In addition, there was not a no intervention control arm; any such an arm would eliminate equipoise in the study, was unlikely to be approved by IRBs given the data available regarding C-BCRL prevention, and there was potential for negative impact upon recruitment and retention, given the level of subject burden for a 3-year study commitment should one group not be offered the intervention. The ALND rate for the study was 17%; this is consistent with modern practice as many patients who previously received ALND will now receive RNI instead.^30^ However, strengths of this study include the design, absence of clinically relevant differences in sociodemographic characteristics between groups before treatment, and length of follow-up.

## Conclusion

Prospective surveillance was conducted over 3-years postoperatively that identified and treated S-BCRL improved patient outcomes. Given the large scale, long-term follow-up, and randomized nature of the trial, these statistically significant results demonstrate that BIS screening should be a standard approach for prospective BCRL surveillance. BIS compared to TM provides a more precise identification of patients likely to benefit from an early compression intervention.
